# Mouse-Adapted H9N2 Influenza A Virus PB2 Protein M147L and E627K Mutations Are Critical for High Virulence

**DOI:** 10.1371/journal.pone.0040752

**Published:** 2012-07-10

**Authors:** Jingjing Wang, Yipeng Sun, Qi Xu, Yuanyuan Tan, Juan Pu, Hanchun Yang, Earl G. Brown, Jinhua Liu

**Affiliations:** 1 Key Laboratory of Animal Epidemiology and Zoonosis, Ministry of Agriculture, College of Veterinary Medicine, China Agricultural University, Beijing, China; 2 Department of Biochemistry, Microbiology and Immunology, Faculty of Medicine, University of Ottawa, Ottawa, Ontario, Canada; 3 Emerging Pathogens Research Centre, Faculty of Medicine, University of Ottawa, Ottawa, Ontario, Canada; 4 The Shandong Animal Disease Control Center, Jinan, China; CSIRO, Australia

## Abstract

H9N2 influenza viruses have been circulating worldwide in multiple avian species and have repeatedly infected humans to cause typical disease. The continued avian-to-human interspecies transmission of H9N2 viruses raises concerns about the possibility of viral adaption with increased virulence for humans. To investigate the genetic basis of H9N2 influenza virus host range and pathogenicity in mammals, we generated a mouse-adapted H9N2 virus (SD16-MA) that possessed significantly higher virulence than wide-type virus (SD16). Increased virulence was detectable after 8 sequential lung passages in mice. Five amino acid substitutions were found in the genome of SD16-MA compared with SD16 virus: PB2 (M147L, V250G and E627K), HA (L226Q) and M1 (R210K). Assessments of replication in mice showed that all of the SD16-MA PB2, HA and M1 genome segments increased virus replication; however, only the mouse-adapted PB2 significantly increased virulence. Although the PB2 E627K amino acid substitution enhanced viral polymerase activity and replication, none of the single mutations of mouse adapted PB2 could confer increased virulence on the SD16 backbone. The combination of M147L and E627K significantly enhanced viral replication ability and virulence in mice. Thus, our results show that the combination of PB2 amino acids at position 147 and 627 is critical for the increased pathogenicity of H9N2 influenza virus in mammalian host.

## Introduction

H9N2 influenza viruses circulate worldwide and are endemic in multiple terrestrial avian species in Asia [1], [2], [3], [4]. It is noteworthy that H9N2 influenza viruses in poultry have occasionally been transmitted to mammalian species, including humans and pigs [5], [6], [7], [8], [9]. Previous studies demonstrated that a significant proportion of H9N2 field isolates have acquired an ability to bind human like receptors [10], [11]. Several serological surveys revealed that a large number of people in China have evidence of prior infections with H9N2 virus especially in poultry workers [12], [13]. Moreover, human H9N2 infections produce a typical human flu-like illness that can easily go undetected [6], [11], [14], providing the viruses a greater opportunity to adapt to humans. These observations raise concerns about the possibility of H9N2 viruses evolving into pandemic strains.

To date, some H9N2 viruses have demonstrated increased virulence for mammals. In 2007 to 2009, Bi et al isolated six H9N2 viruses from chickens in northern China that were highly lethal to mice with properties of systemic spread because they replicated well in multiple organs without prior adaptation [15]. Moreover, the A/Chicken/Hebei/4/2008 virus caused acute respiratory distress syndrome (ARDS) in mice, including diffuse pneumonia and alveolar damage, severe progressive hypoxemia, lymphopenia, and a significant increase in neutrophils [16]. A mouse-adapted H9N2 virus, generated by serial lung-to-lung passage, gained improved growth characteristics on mammalian cells, extended tissue tropism in mice, and was lethal for mice [17]. These studies highlight the necessity for investigation of the genetic basis that determines H9N2 influenza virus host range and pathogenicity in mammals.

The pathogenesis of influenza A viruses is a polygenic trait [18], [19], [20], [21], [22]. PB2 is a particularly well-characterized polymerase gene, and the amino acid at position 627 of the PB2 protein is recognized as a critical mammalian host determinant [23], [24], [25]. PB1 and recently identified PB1-F2 have also been implicated in mouse lung virulence [19], [20], [22], [26]. Isoleucine residue at position 97 in PA protein plays a key role in enhanced virulence in mice and is implicated in the adaptation of avian influenza viruses to mammalian hosts [27]. HA and NA genes are of key importance for host specificity and virulence because they determine specific receptor usage and efficient cell entry, as well as formation and release of progeny virus particles [28]. The M gene also has the capacity to control virulence and replication of mouse-adapted virus [21]. NS1 is a multifunctional protein and is a virulence factor that functions to suppress host innate immune responses [29], [30]. However, most of these studies focused on H1, H3, H5 and H7 subtypes of influenza viruses, and thus research on pathogenic mechanism of H9N2 viruses is lacking.

Mice are ideal animal models for investigating pathogenic mechanisms and host range determinants of influenza A viruses [31], and can be used to generate mouse-adapted variants by serial lung-to-lung passages [17], [32], [33]. Mouse adapted viruses have acquired virulence determining functions, and usually induced pathology in bronchi or lungs of infected mice [34], [35], [36], that is similar to human influenza pneumonia [37]. In the present study, we generated a mouse-adapted H9N2 virus with significantly higher virulence than wide-type virus. Using revere genetics approaches we assessed the differences in wild-type and mouse-adapted variants to identify molecular determinants of host adaptation and virulence of H9N2 viruses in mammals. Our findings suggest that combination of 147L and 627K in PB2 protein is a major contributor to the adaptation and increased virulence of H9N2 influenza virus in mice.

## Materials and Methods

### Ethics statement

All animal research was approved by the Beijing Association for Science and Technology, the approve ID is SYXK (Beijing) 2007–0023, and complied with the guidelines of Beijing laboratory animal welfare and ethical of Beijing Administration Committee of Laboratory Animals.

### Cells and viruses

Human embryonic kidney (293T) and Madin-Darby canine kidney (MDCK) cells (Peking Union Cell Center) were maintained in Dulbecco's modified Eagle's medium (DMEM; Invitrogen) supplemented with 10% fetal bovine serum (FBS).

The H9N2 virus A/chicken/Shandong/16/05 (SD16) was isolated from diseased chicken in Shandong, China and propagated in 10-day-old embryonated chicken eggs (ECE) at 35°C for 72 h. Virus titers were measured by plaque assay in MDCK cells and were expressed as plaque forming units (pfu). All experiments with live viruses and transfectants generated by reverse genetics were carried out in a biosafety level 3 conditions with investigators wearing appropriate protective equipment and compling with general biosafety standard for microbiological and biomedical laboratories of Ministry of Health of the People's Republic of China (WS 233–2002).

### Adaptation of H9N2 virus in mice

Groups of five to seven week-old BALB/c mice (Beijing Experimental Animal Center) were lightly anesthetized with Zoletil 50 (tiletamine-zolazepam; Virbac S.A., Garros, France) and inoculated intranasally with 50 μl of allantoic fluid containing SD16 virus. At 3 days post-inoculation (dpi), mice were euthanized and their lungs were harvested and homogenized, and 50 μl of supernatant from the centrifuged homogenate was used as inoculum for the next passage. After 8 passages, the infected mice died within 6 dpi. Then the virus in the lung homogenate was cloned twice by plaque purification in MDCK cells, and the cloned virus was passaged once in the allantoic cavities of 10-day-old ECE at 35°C for 72 h to prepare a virus stock. The experimental protocol was evaluated and approved by the Animal Pathogenic Microbiology Laboratory Bio-safety Committee, College of Veterinary Medicine, China Agricultural University.

### Sequence analysis

Viral RNAs were extracted from the allantoic fluid of ECE infected with plaque-purified mouse-adapted SD16 (SD16-MA) virus and the eight viral genes were amplified by Reverse transcription-PCR (RT-PCR). The amplified cDNAs were inserted into the genomic expression plasmid PHW2000 [38], which was kindly provided by George F. Gao of Chinese Academy of Sciences, and sequenced.

### Plasmid construction and virus rescue

The eight gene segments of SD16 and SD16-MA were amplified by RT-PCR and cloned in the genomic expression plasmid, PHW2000. Mutations of interest in the PB2 gene were introduced by PCR-based site-directed mutagenesis with primer pairs containing point mutations. All of the constructs were sequenced to confirm the site-directed mutations.

SD16, SD16-MA, reassortant viruses between SD16 and SD16-MA, and SD16 PB2 mutants were generated by reverse genetics as described previously [39]. Briefly, 0.5 µg of plasmid for each gene segment was mixed and incubated with 10 µl of Lipofectamine 2000 (Invitrogen, Carlsbad, CA) at 20°C for 40 min. The Lipofectamine-DNA mixture was transferred to 70% confluent 293T/MDCK cocultured monolayers and incubated at 37°C with 5% CO_2_. Six hours post-transfection, the supernatants were replaced with 2 ml of OPTI-MEM containing 2 μg/ml TPCK-trypsin (Sigma–Aldrich). Forty-eight hours post-transfection, the supernatants were harvested and propagated in 10-day-old ECE to produce stock viruses. Virus titers were determined by plaque assay on MDCK cells.

### Mouse experiments

Groups of 5-week-old female BALB/c mice (Beijing Experimental Animal Center) were anesthetized with Zoletil 50 (tiletamine-zolazepam; Virbac S.A., Carros, France; 20 μg/g) and inoculated intranasally with 10^5^ pfu of viruses in 50 μl phosphate buffered saline (PBS). Three mice in each group were euthanized at 3 dpi, lungs, hearts, livers, spleens, kidneys and brains were collected for virus titration in MDCK cells. The remaining five mice in each group were monitored for weight loss and mortality for 14 days. Mice that lost more than 25% of their body weight were humanely euthanized.

To determine the fifty percent mouse lethal dose (MLD_50_), groups of five 5-week-old female mice anesthetized with Zoletil 50 and inoculated intranasally with 50 μl of 10-fold serial dilutions of viruses in PBS. The mice were monitored for 14 days. MLD_50_ was calculated and expressed in pfu.

For histopathology analysis, mouse lungs collected at 3 dpi were fixed in 10% phosphate-buffered formalin, embedded in paraffin, then cut into 5 μm-thick sections and stained with haematoxylin-and-eosin (H&E).

For cytokine quantification, mouse lungs collected at 3 dpi were stored in single use aliquots at −70°C. Lungs (n = 3) were homogenized individually in 500 μl tissue lysis buffer. Cytokine concentration in lungs were measured using the Bio-Plex Mouse Cytokine 8-Plex panels (Bio-Rad, USA) with beads specific for mouse IL-1β, IL-2, IL-4, IL-5, IL-10, IFN-γ, TNF-α and GM-CSF. Array analysis was performed by the Bio-Plex Protein Array System (Bio-Rad, USA).

### Viral growth kinetics

Confluent MDCK or A549 cells were infected with SD16, SD16-MA or rescued viruses at a multiplicity of infection (MOI) of 0.01, overlaid with serum-free DMEM containing 2 μg/ml TPCK-trypsin (Sigma–Aldrich) and incubated at 37°C. Cell supernatants were harvested every 12 hours until 60 hours post infection (hpi) and titrated by plaque assay on MDCK cells.

### Polymerase activity assay

The PB2, PB1, PA and NP gene segments of SD16, SD16-MA and SD16 PB2 mutants were inserted into pCDNA3.1 plasmid. The PB2, PB1, PA and NP plasmids (125 ng each) were transfected to sixty percent confluent 293T cells, together with fire-fly luciferase reporter plasmid pYH-Luci (10 ng) and internal control plasmid expressing renilla luciferase, Renilla (2.5 ng). After 24 hours of transfection, cell lysate was prepared with Dual-Luciferase Reporter Assay System (Promega) and luciferase activity was measured using GloMax 96 microplate luminometer (Promega).

### Statistical analyses

Statistically significant differences between experimental groups were determined using analysis of variance (ANOVA) with the GraphPad Prism software package (GraphPad Software Inc., La Jolla, CA, USA). A *P*-value less than 0.05 was considered statistically significant.

## Results

### Adaptation of H9N2 influenza virus in mice

Most H9N2 influenza viruses are avirulent for mice [39], [40]. To generate mouse-adapted virus, the H9N2 strain A/chicken/Shandong/16/05 (SD16) was serially passaged in BALB/c mice, beginning with intranasal inoculation of 10^6^ pfu of virus per mouse. Survival of infected animals was monitored and virus titers of lung homogenates were determined after each passage. The mice infected with wild-type SD16 did not show any clinical symptom of disease and virus titer in the lung was 7.3 log_10_ pfu/ml. However, after 5 to 6 passages, virus titers increased to 8.5 to 8.8 log_10_ pfu/ml (data not shown). After 8 passages, mice showed a extensive weight loss and died within 6 dpi, indicating that mouse-adapted variants of H9N2 virus had acquired mutations that profoundly affected virulence.

### Enhanced replication of SD16-MA virus *in vitro*

**Figure 1:**
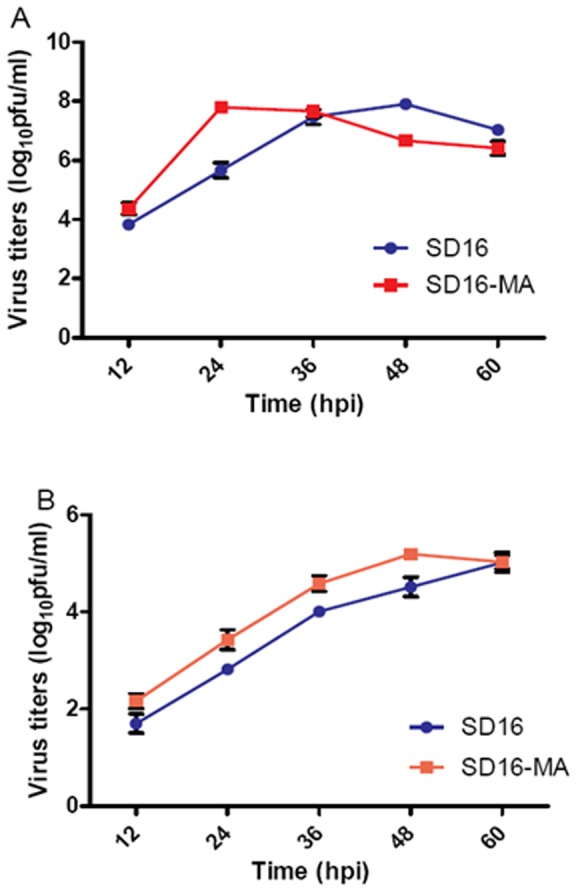
Viral growth kinetics of SD16 and SD16-MA in MDCK and A549 cells. Confluent MDCK (A) or A549 (B) cells were infected with H9N2 viruses at an MOI of 0.01. Virus yields at 12, 24, 36, 48, and 60 hpi were titrated in MDCK cells. Each data point represents the mean virus yield from three individually infected wells ± SD.

To evaluate the replicative ability of mouse-adapted H9N2 virus in vitro, we studied its growth both in MDCK and in A549 cells relative to the wild-type strain. Though the peak titers of the two viruses were similar in MDCK cells (*P*>0.05), the SD16-MA grew faster than SD16, reaching its maximum titers of 7.8 log_10_ pfu/ml at 24 hpi, versus virus titer of 5.7 log_10_ pfu/ml at 24 hpi (*P*<0.05) and reaching peak yield at 48 hpi for SD16 (Fig. 1A). The yields of SD16-MA in A549 cells were 0.12 to 0.64 log_10_ pfu higher than SD16 before 60 hpi (*P*<0.05), while the peak yield of SD16-MA was similar to SD16 (*P*>0.05) (Fig. 1B). These results indicated that SD16-MA grew faster than SD16 both in MDCK and A549 cells, but the peak titers of them were similar.

### Pathogenicity of mouse-adapted H9N2 viruses in mice

**Figure 2:**
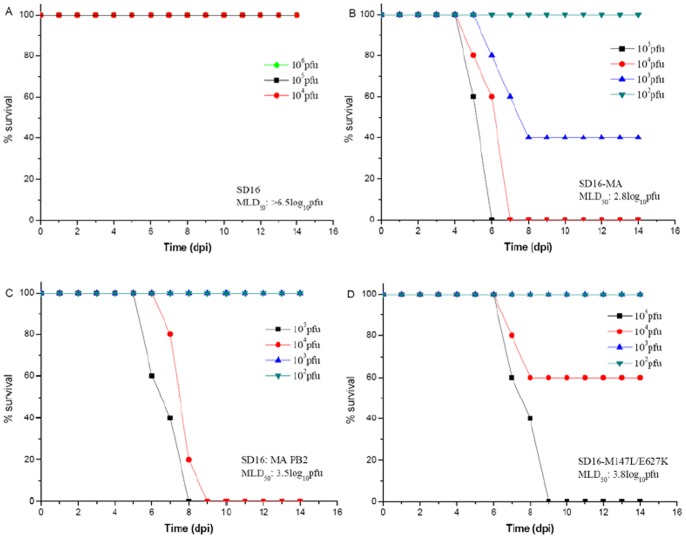
Virulence and death pattern of mice infected with different H9N2 viruses. Five-week-old BALB/c mice (five/group) were inoculated intranasally with different H9N2 viruses, SD16 (A), SD16-MA (B), SD16:MA PB2 (C), or SD16-M147L/E627K (D). Doses of 10^4^ to 10^6^ pfu (A) or 10^2^ to 10^5^ pfu (B, C, D) were used.

**Figure 3:**
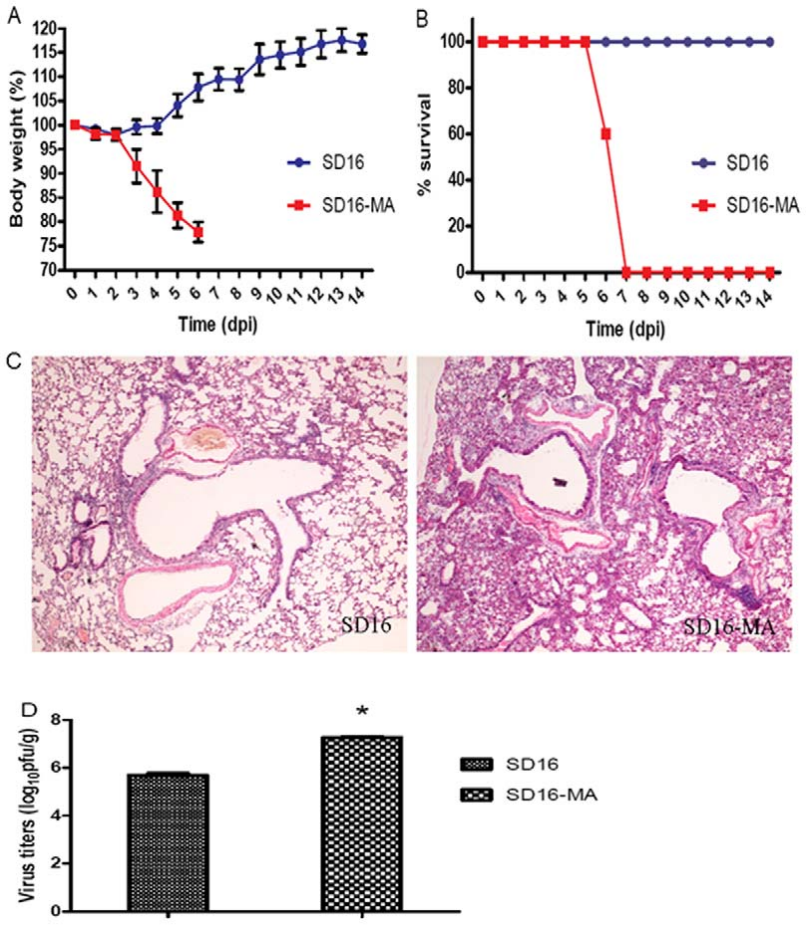
Virulence of SD16 and SD16-MA viruses in mice. Groups of five mice were inoculated intranasally with 10^5^ pfu of SD16 or SD16-MA virus. Body weight (A) and survival (B) were monitored daily for 14 dpi. (C) Histopathology of SD16 and SD16-MA infected mice. Lungs were collected at 5 dpi and fixed in 10% formalin, embedded in paraffin and sectioned. SD16 caused mild and limited alveolitis, whereas SD16 induced severe interstitial pneumonia, including diffuse injury of lungs, alveolar collapse, red blood cells and immune cells infiltration. (D) Virus titers in lungs of infected mice. Groups of three mice were inoculated intranasally with 10^5^ pfu of SD16 or SD16-MA viruses and lungs were collected at 3 dpi for virus titration in MDCK cells. Each value represents mean viral yield from three individually infected lungs ± SD. *, *P*<0.05 compared with the value of SD16.

Using a BALB/c mouse model, we compared the virulence of generated mouse-adapted variant SD16-MA with that of the wild-type SD16 strain. The MLD_50_ value showed that SD16-MA was >10^3.5^-fold more virulent than SD16 virus (Fig. 2A and B). Furthermore, for a given dose of virus (10^5^ pfu), all mice infected with SD16-MA exhibited clinical signs of disease, including decreased activity, huddling, bunched posture, and ruffled fur. Mice in this group lost 20.8% weight and began to die at 6 dpi (Fig. 3A and B). In contrast, no morbidity or mortality was observed in mice infected with 10^5^ pfu of SD16 virus (Fig. 3B). At this dose H&E staining showed that SD16 caused mild and limited alveolitis, whereas SD16-MA induced severe interstitial pneumonia of the infected mice, characterized by diffuse injury of lungs, alveolar collapse, the lung air spaces were filled with red blood cells, immune cells and inflammatory (Fig. 3C).

**Figure 4:**
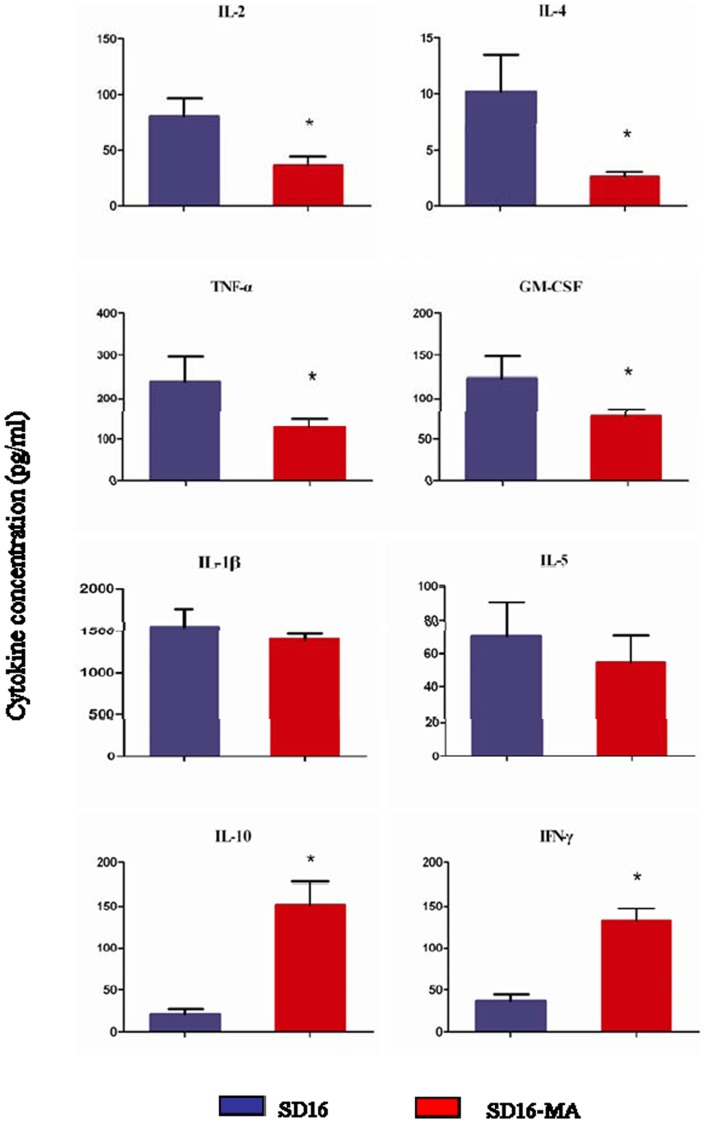
Cytokine responses in lungs of infected mice. Groups of three BALB/c mice were inoculated intranasally with 10^5^ pfu of SD16 or SD16-MA virus. The lungs of mice were collected at 3 dpi. The concentrations of various cytokines in lung homogenates were measured by a protein array analysis with Bio-Plex Mouse Cytokine 8-Plex. Each value represents mean cytokine concentration of three mice from each infected group ± SD. *, *P*<0.05 compared with the value of SD16 infected mice.

Inflammatory cytokines and chemokines have been suggested to be involved in the pathogenesis of animal and human infected with influenza viruses [41], [42], [43]. Cytokines and chemokines IL-1β, IL-2, IL-4, IL-5, IL-10, IFN-γ, TNF-α and GM-CSF in lungs of infected mice were tested at 3 dpi by a protein array analysis with Bio-Plex Mouse Cytokine 8-Plex. As shown in Fig. 4, production of most cytokines resulting from SD16-MA infections was lower than that of SD16 virus (*P*<0.05); IL-1β and IL-5 levels in lungs of mice infected with SD16 and SD16-MA were similar (*P>*0.05); while the levels of IL-10 and IFN-γ in lung homogenates of mice inoculated with SD16-MA were higher than those of SD16 infected mice (*P*<0.05). In summary, no obvious correlation was found between the production of inflammatory cytokines and chemokines and the viral pathogenicity.

To determine whether the differences in virulence of SD16 and SD16-MA were due to different abilities to spread and replicate in mouse organs, groups of three BALB/c mice were euthanized at 3 dpi, and various organs, including lung, brain, heart, liver, spleen, and kidney, were harvested for virus detection and titration. Mouse-adapted H9N2 variant replicated to significantly higher titers in the lungs (up to 7.2 log_10_ pfu/g, *P*<0.05) than wild-type SD16 virus (∼5.7 log_10_ pfu/g) (Fig. 3D). Both viruses were not detected in other organs (<50 pfu/g). Taken together, SD16-MA had greater pathogenicity and replication advantage over SD16 virus, but could not spread and replicate systemically in mice.

### Five amino acid substitutions emerged after adaptation of SD16 to mice

**Figure 5:**
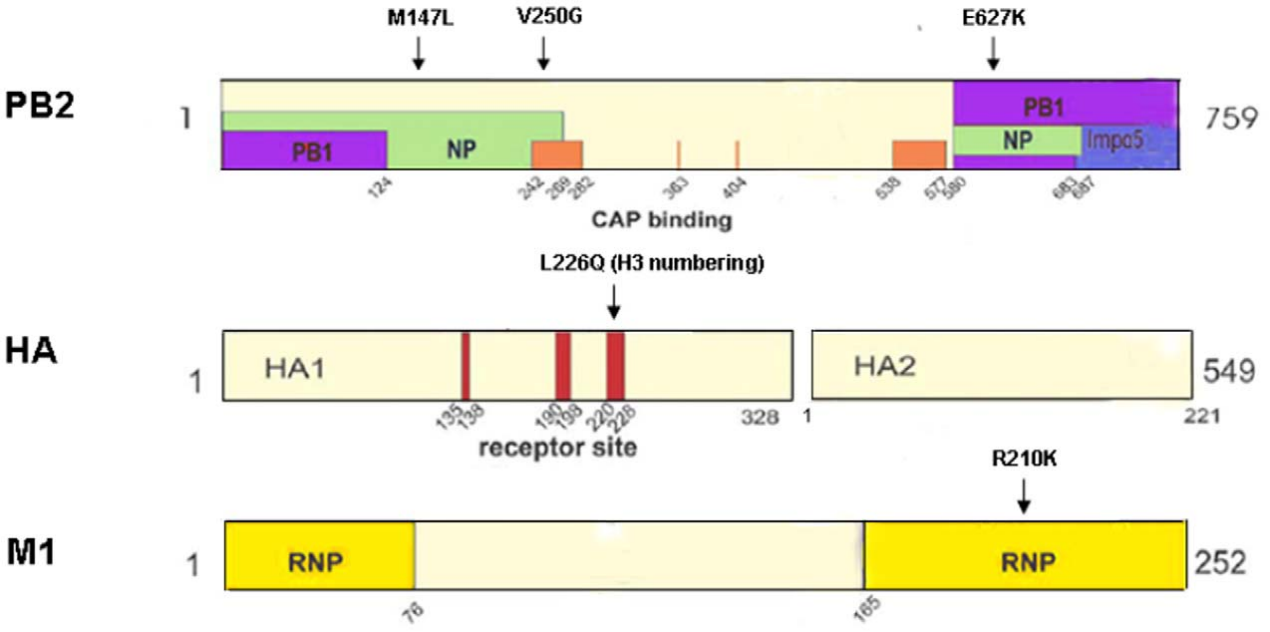
Amino acid differences between SD16 and SD16-MA. The amino acid location of mutations are numbered and indicated with arrowheads on the linear sequence. The locations of regions of protein binding, or functions are indicated with rectangles and are labeled with respect to interacting viral proteins. The PB1, NP and RNP ribonucleocapsid protein binding regions are in purple, green and yellow respectively; PB2 cap binding regions are in orange; HA receptor sites are in red.

To identify the molecular markers responsible for the virulence of mouse-adapted variant in BALB/c mice, the genomes of the wild-type and mouse-adapted virus were sequenced. Sequence analysis identified five mutations among 3 genes that were involved in adaptation of SD16 strain to mice; specifically PB2 (M147L, V250G, and E627K), HA (L226Q), and M (R210K) (Fig. 5). The PB2 M147L mutation resided in the N-terminal NP binding region (1–269aa) and PB2 V250G mapped to a site involved in the host 7methyl guanosine cap binding domain (242–282aa) and NP binding region [32]. PB2 E627K is as a host range determinant that determines the pathogenicity of H5N1 influenza viruses in mice [44]. The L226Q (H3 numbering) mutation in HA occurred the receptor-binding site (RBS). The M1 R210K mutation was in a region (165–252aa) that had been shown to bind ribonucleoprotein (RNP) [32]. These results indicated that the mutations observed in SD16-MA may be related to various regulatory functions that control influenza virus infection and replication.

### Mutations in SD16-MA PB2 modulate polymerase activity

**Figure 6:**
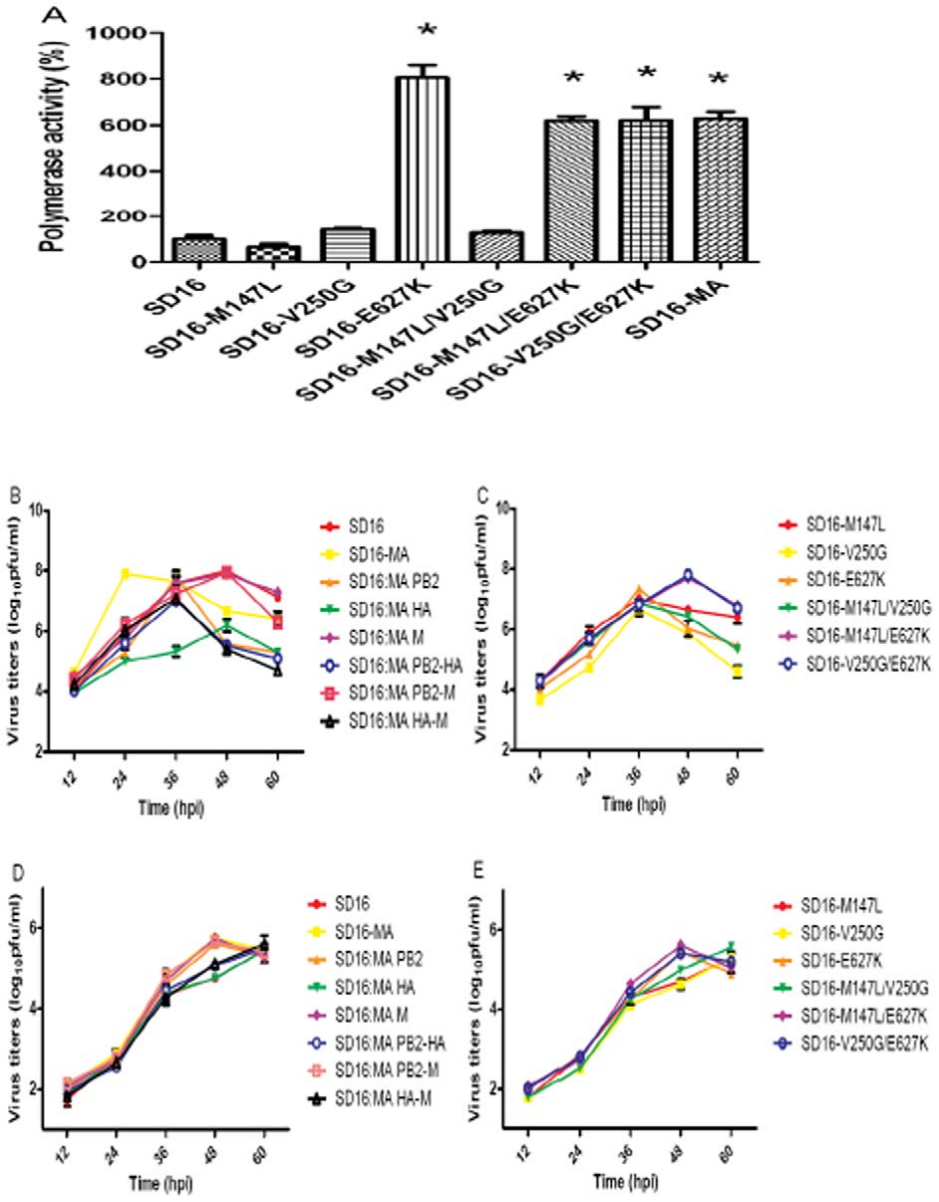
Viral RNA polymerase activity and Viral growth kinetics in MDCK and A549 cells. (A) Polymerase activity of SD16 with different PB2 mutations in a minigenome assay. Four protein expression plasmids (PB2, PB1, PA, NP) for the RNP combinations were transfected into 293T cells together with luciferase reporter plasmid pYH-Luci and internal control plasmid Renilla, as described in Materials and Methods. The values shown are means ± SD of results for three independent experiments and are standardized to the activity of SD16 (100%). *, *P*<0.05 compared with that of SD16-infected cells. (B, C, D, E) Viral growth kinetics of reassortant viruses and PB2 mutants in MDCK and A549 cells. Confluent momolayers of cells were infected with rescued H9N2 viruses at an MOI of 0.01. Cell supernatants were harvested every 12 hours until 60 hpi and titrated by plaque assay on MDCK cells. Each data point represents the mean virus yield from three individually infected wells ± SD. (B) reassortant viruses in MDCK cells, (C) PB2 mutants in MDCK cells, (D) reassortant viruses in A549 cells, (E) PB2 mutants in A549 cells.

To evaluate whether the observed mutations in the PB2 protein of mouse-adapted H9N2 variant affect polymerase activity, we measured the activity of reconstituted RNP complexes in 293T cells by a luciferase minigenome assay. SD16-MA carrying PB2 M147L, V250G, and E627K had polymerase activities that was ∼630% relative to wild-type SD16 virus (100%) (Fig. 6A). To assign this increase to single mutations, polymerase activities of all possible combinations were examined. PB2 E627K mutation increased polymerase activity of SD16 RNP complex to ∼805%, whereas M147L and V250G did not significantly increase polymerase activity as PB2 E627K. In combination the M147L and V250G only possessed a marginally increased polymerase activity to ∼127% that of SD16. In contrast, the combination of E627K plus M147L, and E627K plus V250G enhanced polymerase activity to ∼620% and 622% respectively, indicating an attenuating effect of the mutations in combination with E627K. Collectively, our findings showed that efficient polymerase activity (>6-fold increase, *P*<0.05) was ∼achieved by PB2 E627K mutation that was similar for all mutant combinations that contained this mutation, PB2 E627K/M147L, PB2 E627K/V250G and PB2 E627K/M147L/V250G.

### Mutations in SD16-MA PB2 and M modulate growth kinetics *in vitro*

To determine the contribution of each SD16-MA mutational gene and SD16-MA PB2 mutation to viral replication *in vitro*, six single or double gene substituted viruses and six PB2 mutants were generated using the SD16 backbone. The inherent growth characteristics of these rescued viruses in MDCK and A549 cells were studied. Although the SD16-MA virus replicated faster in MDCK cells than SD16, none of the SD16 viruses containing SD16-MA genome segments grew to higher titers than the SD16 parental strain in MDCK cells. Whereas many reassortants generated lower yields than SD16, those reassortants containing SD16-MA PB2 and/or M genes replicated to the highest yields that were better than other single or double gene reassorted viruses (*P*<0.05), with the maximum titers ranging from 7.80–7.95 log_10_ pfu/ml (Fig. 6B). For the six mutated viruses, SD16-M147L/E627K and SD16-V250G/E627K grew more efficiently than the other four viruses (*P*<0.05), reaching maximum titers of more than 7.7 log_10_ pfu/ml at 48 hpi (Fig. 6C) but none generated higher tires than SD16 virus. Moreover, the viruses that replicated well in MDCK cells all grew faster in A549 than the other viruses, and reached their peak titers at 48 hpi, a time 12 hours faster than that for the other viruses (Fig. 6D to E). These results suggested that M1 R210K, PB2 147L/627K and 250G/627K combination could enhance viral replication *in vitro*.

### Mutations in PB2 contribute to the virulence of SD16-MA

To determine the role of each SD16-MA mutational gene in the virulence phenotype, six reassortant viruses (Table 1) were generated using the SD16 backbone and their virulence was evaluated in BALB/c mice. Though introduction of genes from SD16-MA significantly improved the replication ability of H9N2 viruses in mice, not all of reassortant viruses gained higher virulence than SD16 virus. Virus containing SD16-MA PB2 gene alone caused significant mortality in mice (Fig. 2C), while SD16-MA HA and M gene could not detectably enhance viral pathogenicity, they did significantly enhance replication in mice lungs (Table 1). Moreover, double gene substitute viruses SD16:MA PB2-HA (MLD_50_  = 10^4.5^ pfu) and SD16:MA PB2-M (MLD_50_  = 10^3.6^ pfu) were less virulent than SD16:MA PB2 (MLD_50_  = 10^3.5^ pfu), indicating negative gene interaction effects for the combinations. These results indicated that mutations in PB2 alone were responsible for the greatest increases in replication and lethality of H9N2 virus in mice.

**Table 1:** Replication and pathogenicity of reassortant viruses between SD16 and SD16-MA in mice.

Virus	3dpi. mean virus titer^a^ (log_10_pfu/g±SD)	Mortality (%)	MLD_50_ (log_10_pfu)
SD16	3/3^b^ (5.5±0.07)	0	>6.5
SD16-MA	3/3 (7.2±0.12)	100	2.8
SD16:MA PB2	3/3 (7.0±0.21)	100	3.5
SD16:MA HA	3/3 (6.2±0.08)	0	>6.5
SD16:MA M	3/3 (7.3±0.24)	0	>6.5
SD16:MA PB2-HA	3/3 (6.6±0.07)	100	4.5
SD16:MA PB2-M	3/3 (7.7±0.38)	100	3.6
SD16:MA HA-M	3/3 (6.7±0.18)	0	>6.5

a Five-week-old female BALB/c mice were inoculated intranasally with 10^5^ pfu of viruses. Three mice from each group were killed at 3dpi. and virus titers in lung were determined.

b indicats the number of mice infected per number of mice inoculated.

### The 147L/627K combination of PB2 mutations was the major contributor to the virulence of SD16-MA

To pinpoint the contribution of each amino acid mutation in PB2 to the virulence of SD16-MA, single and double mutants were generated using the remaining 7 genes from SD16. MLD_50_ and viral replication in lungs of these viruses in BALB/c mice were evaluated. Single amino acid substitution PB2 M147L or V250G did not increase the pathogenicity of SD16 in mice with MLD_50_ >10^6.5^ pfu, while PB2 E627K slightly increased pathogenicity with MLD_50_ reduced more than 10-fold compared to wild-type virus (Table 2). Among the six mutants, only SD16-M147L/E627K virus had similar virulence (MLD_50_  = 10^3.8^ pfu ) to SD16:MA PB2 (MLD_50_  = 10^3.5^ pfu) (Fig. 2C and D), indicating that the 147L/627K combination is the major contributor to virulence imparted by PB2 of mouse adapted H9N2 virus. Moreover, lung virus titer for SD16-M147L/E627K was approximately 0.7 and 0.8 log_10_ pfu higher than SD16-147L and SD16-627K, respectively, and approximately 1.4 log_10_ pfu higher than SD16 virus (Table 1 and 2). Thus, these single and double mutants clearly demonstrated that only the combination of PB2 M147L and E627K could increase the virulence of the H9N2 virus in BALB/c mice.

**Table 2:** Replication and pathogenicity of mutants in mice.

Virus	PB2			3dpi. mean virus titer^a^ (log_10_pfu/g±SD)	MLD_50_ (log_10_pfu)
	147	250	627		
SD16	M	V	E	3/3^b^ (5.5±0.07)	>6.5
SD16-M147L	**L**	V	E	3/3 (6.2±0.18)	>6.5
SD16-V250G	M	**G**	E	3/3 (5.5±0.22)	>6.5
SD16-E627K	M	V	**K**	3/3 (6.1±0.09)	5.5
SD16-M147L/V250G	**L**	**G**	E	3/3 (6.7±0.08)	5.7
SD16-M147L/E627K	**L**	V	**K**	3/3 (6.9±0.04)	3.8
SD16-V250G/E627K	M	**G**	**K**	3/3 (6.5±0.07)	5.5

a Five-week-old female BALB/c mice were inoculated intranasally with 10^5^ pfu of viruses. Three mice from each group were killed at 3dpi and virus titers in lung were determined.

b number of mice infected/number of mice inoculated.

## Discussion

Viral adaptation is considered to be one of the major mechanisms for generating pandemic influenza viruses [45]. However, the molecular basis of adaptation of influenza A virus to a new host is poorly understood. The present results showed that adapted virus could be obtained by serial passages and PB2 gene of SD16-MA is critical for the virulence of mouse-adapted H9N2 influenza virus, especially, the combination of PB2 M147L and E627K contribute to the highly lethality of mouse-adapted H9N2 influenza virus.

In the study, viruses containing SD16-MA PB2, HA or M gene all grew better in lungs of mice than SD16, whereas mutations in PB2 and M1 protein demonstrated increased viral replication ability *in vitro*, but only the mouse-adapted PB2 gene significantly increased viral virulence. Thus all of the 3 mutant genes were demonstrated to possess increased replicative functions in mice indicating the functional basis for their selection on mouse adaptation. The PB2 gene has multiple functions, such as binding host capped mRNAs and initiating viral mRNA synthesis, making it an important pathogenic determinant [46], [47], [48]. The amino acid at position 627 of the PB2 protein has been described as a host range determinant, and the mutation E627K has been shown to be a key factor in the adaptation of H5N1 [23], [24], [25] or other avian subtypes [49], [50] influenza viruses to mammals. Hossain *et al* adapted a wildtype duck H9N2 virus in quail and chickens through serial lung passages. The adapted viruses were readily infect mice and resulted in a quick selection of PB2 E627K mutation [51]. It has also been demonstrated that viruses with lysine at this position were lethal in mice, whereas those with glutamic acid were avirulent [44]. However, in our study, PB2 E627K mutation did not significantly increase pathogenicity though it could increase viral replication in lungs of mice, indicating that the viral genomic backbone affects the phenotype of mutations at position 627. Herfst et al also found that introduction of E627K mutation to H1N1/2009 virus had no major impact on virus replication in the respiratory tracts of mice and ferrets or on pathogenesis [52]. Another report showed that PB2 E158G could increase the morbidity and mortality of the H1N1 pandemic virus, which was much stronger than the effect of PB2 E627K [53]. All these data suggested that the virulence function of PB2 E627K seemed to be strain-specific or needed to interact with residue at other positions.

Most of the previous studies found major contributions of single amino acid mutations to viral adaptation and virulence, such as PB2 E158G [53], I504V [28], E627K [54], D710N [32], here, we demonstrated the importance of synergism of two or more amino acid substitutions in combination with E627K. Liu et al also found that a combination of PB2 271A with 590/591 SR polymorphism was critical for H1N1/2009 and triple reassortant swine influenza viruses for efficient replication and adaptation in mammals [55]. In the present study, introducing single mutation of M147L, V250G or E627K to SD16 PB2 protein did not significantly increase the pathogenicity of virus in mice, nevertheless, substitution of two amino acid residues could increase the virulence, among which the 147L/627K combination caused significant mortality. Both PB2 147 and 627 positions were located in PB2 and NP interaction regions, where mutations at these two positions might complement each other and mediate enhanced protein function, leading to increased replication and mortality. However, this explanation needs to be further investigated by crystal structural determination of PB2-NP and possibly host protein complexes. The structure of C-terminal domain of influenza virus polymerase PB2 subunit has been solved to show that K627 is located on a large solvent-exposed face of the protein with a predominant positively charged surface [56]. Nevertheless, the structure of N-terminal domain of PB2 protein is not known. So, the exact relationship of amino acids between position 147 and 627 in PB2 protein awaits further investigation.

Increased polymerase activity was considered to play a role in the adaptation of influenza viruses, because it corresponded to the optimal growth in mammalian cells, replicative fitness and virulence in mice [27], [28], [33], [53]. However, some mutations in the polymerase genes acted to enhance the polymerase activity, but not pathogenicity of virus [32], [57]. Song *et al* reported that PA N383D contributed to the difference in the polymerase activities of two H5N1 viruses, but could not increase viral virulence, which may be affected by high level of polymerase accumulation in the nucleus of influenza virus-infected cells. Here, PB2 E627K increased the polymerase activity and replication of virus, but did not significantly increase morbidity, as did the combination of V250G and E627K. Combining mutations at 147 and 627 in PB2 could confer increasing polymerase activity and virulence to the adapted virus. These results suggested that high polymerase activity was a prerequisite for the high virulence.

Viral replication is thought to be an important and characteristic prerequisite for virulence. Some highly pathogenic H5N1 avian influenza viruses and wild-type H9N2 viruses that were lethal to mice without adaptation all replicated well in lungs, and even replicated systemically [15], [58], [59]. The SD16 virus, used in this study, was avirulent to mice and only could be detected at lower titers in the lung of infected mice. After adaptation, SD16-MA virus gained higher virulence and replicative fitness in mice with major roles shown for M147L/E627K mutations that possessed higher virulence than SD16, accompanied by greater virus titers in lungs of mice. The SD16-M147L, SD16-E627K, SD16-V250G/E627K mutants also replicated efficiently *in vivo* and *in vitro*, but were not lethal for mice. These results suggested that high virus load was a prerequisite for the high virulence of virus in mice, but not sufficient. Other factors, such as polymerase activity and regulation of the proinflammatory response, also contribute to virulence of influenza viruses [27], [60]. Here, we observed that levels of IL-10 and IFN-γ in lungs of SD16-MA infected mice were higher than those induced by SD16 virus. The elevated levels of IL-10 and IFN-γ were also found in the peripheral blood of patients infected with H5N1 influenza virus [61]. IL-10 plays an important role in the dampening of inflammatory response to prevent excessive damage to the host [62]. IFN-γ is known to mediate the increased production of nitric oxide [63], which can subsequently result in the recruitment of more neutrophils and macrophages [64].

In summary, using a highly lethal mouse-adapted H9N2 virus, we demonstrated mutations in PB2 protein were critical for the adaptation and virulence of H9N2 virus in mice. Although the E627K mutation on its own enhanced replication and polymerase activity, it did not significantly increase pathogenicity of virus in mice. The combination of M147L and E627K in PB2 was critical for the high virulence of mouse-adapted H9N2 virus.
